# Supplementary material for: Molecular Study of Pneumocystis jirovecii in Respiratory Samples of HIV Patients in Chile
Source: J Fungi (Basel). 2024 Jan 31;10(2):117. doi: 10.3390/jof10020117 (PMC10889964; doi:10.3390/jof10020117)
Supplement: Supplementary file 1 [file jof-10-00117-s001.zip › jof-2612815-supplementary.pdf]

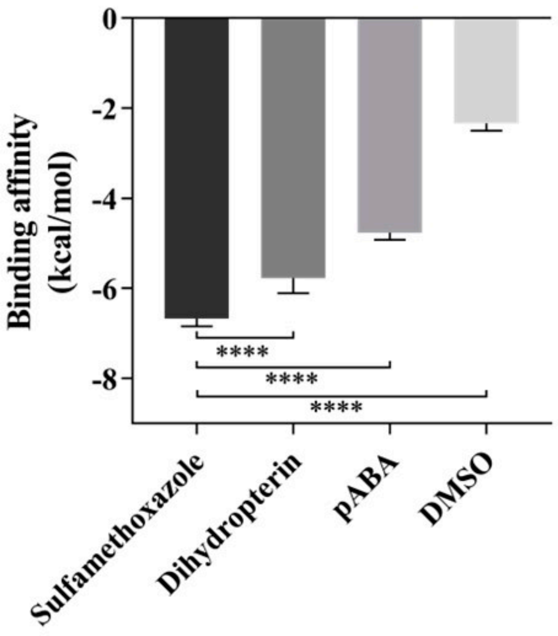

Figure S1. Docking analyses involving the wild type enzyme model of dihydropteroate synthase with sulfamethoxazole, dihydropterin, PABA, and DMSO. Significant differences are indicated by asterisks (****), which denote the level of significance ($p < 0.05$).
